# Ti_3_C_2_-MXene/NiO Nanocomposites-Decorated CsPbI_3_ Perovskite Active Materials under UV-Light Irradiation for the Enhancement of Crystal-Violet Dye Photodegradation

**DOI:** 10.3390/nano13233026

**Published:** 2023-11-27

**Authors:** Asma A. Alothman, Mohammad Rizwan Khan, Munirah D. Albaqami, Sonaimuthu Mohandoss, Zeid A. Alothman, Naushad Ahmad, Khadraa N. Alqahtani

**Affiliations:** 1 Department of Chemistry, College of Science, King Saud University, Riyadh 11451, Saudi Arabia; aaalothman@ksu.edu.sa (A.A.A.); zaothman@ksu.edu.sa (Z.A.A.);; 2 School of Chemical Engineering, Yeungnam University, Gyeongsan 38541, Republic of Korea; drsmohandoss@yu.ac.kr

**Keywords:** Ti_3_C_2_-MXene, NiO, perovskite, crystal violet, UV-light irradiation, photodegradation

## Abstract

Ti_3_C_2_-MXene material, known for its strong electronic conductivity and optical properties, has emerged as a promising alternative to noble metals as a cocatalyst for the development of efficient photocatalysts used in environmental cleanup. In this study, we investigated the photodegradation of crystal-violet (CV) dye when exposed to UV light using a newly developed photocatalyst known as Ti_3_C_2_-MXene/NiO nanocomposite-decorated CsPbI_3_ perovskite, which was synthesized through a hydrothermal method. Our research investigation into the structural, morphological, and optical characteristics of the Ti_3_C_2_-MXene/NiO/CsPbI_3_ composite using techniques such as FTIR, XRD, TEM, SEM–EDS mapping, XPS, UV–Vis, and PL spectroscopy. The photocatalytic efficacy of the Ti_3_C_2_-MXene/NiO/CsPbI_3_ composite was assessed by evaluating its ability to degrade CV dye in an aqueous solution under UV-light irradiation. Remarkably, the Ti_3_C_2_-MXene/NiO/CsPbI_3_ composite displayed a significant improvement in both the degradation rate and stability of CV dye when compared to the Ti_3_C_2_-MXene/NiO nanocomposite and CsPbI_3_ perovskite materials. Furthermore, the UV–visible absorption spectrum of the Ti_3_C_2_-MXene/NiO/CsPbI_3_ composite demonstrated a reduced band gap of 2.41 eV, which is lower than that of Ti_3_C_2_-MXene/NiO (3.10 eV) and Ti_3_C_2_-MXene (1.60 eV). In practical terms, the Ti_3_C_2_-MXene/NiO/CsPbI_3_ composite achieved an impressive 92.8% degradation of CV dye within 90 min of UV light exposure. We also confirmed the significant role of photogenerated holes and radicals in the CV dye removal process through radical scavenger trapping experiments. Based on our findings, we proposed a plausible photocatalytic mechanism for the Ti_3_C_2_-MXene/NiO/CsPbI_3_ composite. This research may open up new avenues for the development of cost-effective and high-performance MXene-based perovskite photocatalysts, utilizing abundant and sustainable materials for environmental remediation.

## 1. Introduction

Many environmental concerns are currently occurring as a result of the impact of many natural and man-made elements on the Earth’s crust [1]. Environmental pollution, in general, refers to any undesirable and unacceptable alterations in the environment resulting from various human activities. These alterations can manifest as direct or indirect changes in the biological, chemical, and physical characteristics of natural water bodies, leading to detrimental effects on both human populations and aquatic ecosystems [2]. Various factors contribute to the pollution of natural water bodies, including rapid population growth, urbanization, and extensive industrialization. One significant source of water contamination is azo dyes, which pose a considerable threat to the environment due to their non-biodegradable and hazardous nature [3]. Azo dyes, a type of dye with diverse applications, account for more than 50% of global dye production. Synthetic azo dyes find extensive use in textiles, food, cosmetics, lithography, and certain medical products [4]. Additionally, they play a crucial role in various technological applications, such as photonics devices, laser dyes, photovoltaics, and antidiabetic drugs. Azo dyes consist of diazotized amines attached to amines or phenols, often containing one or more azo linkages. The precursor compounds of azo dyes are aromatic amines. Some azo compounds exhibit remarkable stability and persist in the environment for extended periods, making them resistant to removal from wastewater through conventional methods. There are several ways to break down dyes in wastewater, including chemical, biological, and physical techniques. Nevertheless, a lot of these methods have significant deterioration and upkeep expenses. Moreover, several of these techniques produce secondary waste products that need to be treated further, making them inappropriate and expensive for treating wastewater [5].

Nanotechnology, on the other hand, has gained a tremendous impetus in this quickly rising technological period by producing an abundance of scientific concepts to compete with the daily problems of growing technology. Nanomaterials have garnered immense interest due to their myriad applications and unique properties, which arise from their distinct size, shape, and surface-area characteristics [6]. One such nanomaterial, MXene, represents a novel class of 2D materials derived from the etching of Ti_3_C_2_, a transition metal carbide, nitride, or carbonitride. MXene has drawn considerable attention as a promising cocatalyst material in the development of heterostructure systems, particularly for photocatalysis [7]. This heightened interest can be attributed to MXene’s remarkable attributes, including its structural stability, an abundance of hydrophilic functional groups (e.g., -O and -OH) on its surface, excellent metal conductivity, and enhanced redox reactivity emanating from its terminal Ti sites. Utilized as a carrier substrate, Ti_3_C_2_-MXene serves a dual purpose. It prevents the agglomeration of nanoscale photocatalysts and effectively captures photoexcited electrons, thus promoting the separation of electron–hole pairs during the photocatalytic process. Nonetheless, the self-stacking tendency of Ti_3_C_2_-MXene sheets can lead to undesirable outcomes, such as reduced surface area and diminished active accessible sites [8]. This self-stacking phenomenon results in a transition of Ti_3_C_2_-MXene properties from metallic to semiconducting. To address this challenge, Ti_3_C_2_-MXene materials are viewed as valuable auxiliary components that can modify the conductivity of active materials when integrated into devices alongside other substances like metal oxides. Consequently, 2D Ti_3_C_2_-MXene materials hold promise as cocatalysts, enhancing the photocatalytic performance of photocatalysts by effectively promoting the separation of photogenerated carriers [9].

Metal oxide semiconductors perform better when two different semiconductors with different photogenerated electron–hole pair energy levels are connected because of their interfacial activity [10]. Nonetheless, one significant challenge associated with these nanomaterials is their propensity to aggregate into secondary particles, which significantly limits their catalytic performance in various applications. Notably, a p–n junction can be formed at the interface between p-type and n-type binary semiconductor oxides, which effectively helps to separate electron–hole pairs [11]. Among the several p-type oxides, nickel oxide (NiO) is a particularly active compound with a broad band gap between 3.6 and 4.0 eV. For a variety of uses, such as chemical sensors, photovoltaic devices, gas sensing, catalysis, magnetic materials, electrochromic films, and battery cathodes, it has been thoroughly investigated [12]. Research indicates that nanoscale materials can exhibit novel and unique properties, and, among them, semiconductor oxides belonging to the group of photocatalyst nanomaterials, such as Fe_3_O_4_, NiO, TiO_2_, and ZnO, hold significant potential for advanced oxidation processes in the context of environmental pollution remediation [13]. While some researchers have investigated the synthesis methods and characteristics of NiO nanoparticles, there is a noticeable lack of reports regarding their functionality as photocatalysts for dye degradation and an examination of the factors influencing photocatalytic degradation in the available scientific literature.

Perovskite materials had previously only been used in semiconducting applications, but their photocatalytic activity and capacity to actively break down constituent particles were investigated for water-refining applications [14]. In recent times, perovskite-based catalysts have piqued the interest of researchers due to their versatile bandgap adjustability, high stability, rapid mobility of photoinduced electrons and holes (e^−^/h^+^), and exceptional photocatalytic activity [15]. In particular, lead trihalide perovskites have become a fascinating family of materials with great potential for applications in the next generation. Superior optical qualities, a high-attenuation coefficient, a configurable bandgap, adaptable surface chemistry, long-range electron–hole diffusion, and high carrier mobility are just a few of their impressive features [16]. These materials typically adhere to the general formula ABX_3_, with A representing a cation (organic or inorganic), B as a divalent metal (Pb^2+^, Sn^2+^, Ge^2+^), and X as an anion (Cl^−^, Br^−^, I^−^, or a combination thereof). Semiconductor materials are widely used as photocatalysts in the energy and environmental domains because of their low cost and special physiochemical properties [17]. The use of inorganic lead trihalide perovskites, their derivatives, and composites as photocatalysts has been the subject of multiple reports in recent times. It has been noted that, among the lead halides based on cesium, pure iodide-based compounds with a broad bandgap provide difficulties for photocatalysis [18,19,20]. However, various treatments, such as creating heterostructures or modifying typical ligands, can render them optimal choices. To the best of our knowledge, there has been no prior research conducted on the utilization of Ti_3_C_2_-MXene/NiO nanocomposite-decorated CsPbI_3_ perovskite and its photocatalytic activity in degrading crystal-violet (CV) dye. Ti_3_C_2_-MXene/NiO/CsPbI_3_ is an effective passivator in three main ways, according to systematic experimental results: (i) it can modify the energy levels of perovskite materials and create a hole-transfer pathway that is efficient; (ii) it can passivate defects and lessen nonradiative recombination at the interface; and (iii) it forms a barrier layer that keeps water out and improves the stability of CsPbI_3_ materials.

The amalgamation of halide perovskites and MXene materials in various configurations represents a cutting-edge frontier in photocatalysis for environmental remediation. The novel insights into the construction of halide perovskite-based photocatalysts, exploring enhanced properties through composites, mechanochemical synthesis mechanisms, and innovative heterostructure designs [21,22,23]. Additionally, the integration of MXene materials into composite electrodes for supercapacitors showcases a breakthrough in energy-storage technologies. The interplay between perovskite structures, such as LaNiO_3_ and MnTiO_3_, and their synergistic interactions with other materials reveal promising advancements in the photocatalytic degradation of pollutants, providing a foundation for sustainable and efficient environmental solutions. This literature presents an innovative approach that combines different nanomaterials, such as Ti_3_C_2_-MXene, NiO, and CsPbI_3_ perovskite, to create a composite material. The focus of the study lies in exploring the photocatalytic capabilities of these materials, particularly in the degradation of crystal-violet (CV) dye in an aqueous solution under UV-light exposure. The Ti_3_C_2_-MXene/NiO/CsPbI_3_ composite exhibits exceptional photocatalytic performance, leading to significant CV dye degradation. The paper delves into the degradation pathway and elucidates the mechanisms involved in the photocatalytic process, emphasizing the composite’s efficiency. Factors contributing to this efficiency include trapping sites for electrons, hindrance of electron–hole pair recombination, a larger surface area, and a lower recombination rate. Therefore, the study provides valuable insights into the design and application of composite nanomaterials for advanced photocatalysis, suggesting potential implications for environmental pollution remediation and diverse future applications.

Herein, for the first time, Ti_3_C_2_-MXene/NiO nanocomposites with CsPbI_3_ perovskite materials were successfully synthesized through the hydrothermal method and characterized by FTIR, XRD, TEM, SEM–EDS mapping, XPS, UV–Vis, and PL techniques. The photocatalytic capabilities of three different materials, namely Ti_3_C_2_-MXene, Ti_3_C_2_-MXene/NiO, and Ti_3_C_2_-MXene/NiO/CsPbI_3_ composites, were assessed by their ability to degrade crystal-violet (CV) dye in an aqueous solution when exposed to UV light. In comparison to the other catalysts, the Ti_3_C_2_-MXene/NiO/CsPbI_3_ composite exhibited a highly advantageous photocatalytic performance, achieving an impressive 92.8% degradation of the CV dye within just 90 min of UV-light exposure. The effectiveness of the Ti_3_C_2_-MXene/NiO/CsPbI_3_ composite can be attributed to its ability to provide ample trapping sites for electrons, which, in turn, hinders the recombination of electron–hole pairs and contributes to the enhanced removal of CV dye through photocatalysis. The paper also delves into the degradation pathway of CV dye and the mechanisms involved in the photocatalytic process. In this context, the rapid and efficient degradation of dye molecules is achieved through a combination of factors, including the larger surface area and a lower electron–hole recombination rate. These characteristics make the Ti_3_C_2_-MXene/NiO/CsPbI_3_ composites highly efficient photocatalysts and promising candidates for a wide range of future applications.

## 2. Materials and Methods

### 2.1. Materials

Ti_3_AlC_2_ powder (approximately 400 mesh), hydrofluoric acid (HF, 40% weight), CsI, PbI_2_, n-butyl acetate, and dimethyl sulfoxide (DMSO) were procured from Sigma-Aldrich (St. Louis, MO, USA). NiSO_4_·6H_2_O and NaOH were obtained from TCI Chemical Reagents. All chemical reagents employed in this study were of analytical grade and were used without any additional purification. Deionized water served as the solvent in all synthesis procedures.

### 2.2. Preparation of Ti_3_C_2_-MXene Nanosheets

In accordance with the methodology outlined in a prior study [24], Ti_3_C_2_-MXene nanosheets were prepared by selectively etching the aluminum (Al) layer from Ti_3_AlC_2_. The Ti_3_AlC_2_ powder was immersed in a concentrated hydrofluoric acid (HF) solution with a concentration of 50% by weight. This immersion was conducted at room temperature and lasted for 24 h, facilitating the removal of the aluminum (Al) atoms from the Ti_3_AlC_2_ structure. The resulting suspension was transferred into a 45 mL centrifuge tube, followed by centrifugation at 3500 rpm for 5 min. This step was performed to separate the etched Ti_3_C_2_-MXene nanosheets from the solution. The centrifuged material was then washed with deionized (DI) water five times to remove any residual HF acid. Subsequently, 0.2 g of the Ti_3_C_2_-MXene powder were combined with 15 mL of dimethyl sulfoxide (DMSO). This mixture was subjected to magnetic stirring for 24 h at room temperature. The final product obtained after the etching process was centrifuged again, this time at 10,000 rpm for 30 min, and then washed with DI water. The washed material was further subjected to vacuum drying to obtain the delaminated Ti_3_C_2_-MXene powder for subsequent use.

### 2.3. Preparation of Ti_3_C_2_-MXene/NiO Composite

The synthesis of the Ti_3_C_2_-MXene/NiO composite was achieved through a hydrothermal method [25]. Initially, 200 mg of the previously prepared Ti_3_C_2_-MXene and 150 mg of NiSO_4_·6H_2_O were dispersed in a solution of 50 mL of NaOH. Ultrasonication was applied for 30 min to ensure effective mixing, followed by continued magnetic stirring. The resulting mixture was then sealed within an 80 mL Teflon-lined autoclave and maintained at a temperature of 150 °C for a duration of 12 h. The product obtained after the hydrothermal treatment was a black slurry. It was subjected to filtration and washed with deionized water five times to remove any impurities. Finally, the resulting material was dried in a vacuum oven at 60 °C for 24 h, leading to the formation of the Ti_3_C_2_-MXene/NiO composite. For comparative purposes, pure NiO was also synthesized using the following abbreviated procedure. A dropwise addition of 50 mL of NaOH solution was made to 15 mL of NiSO_4_·6H_2_O. The mixture was stirred for 2 h, and, as a result, a precipitate of Ni(OH)_2_ was obtained. The Ni(OH)_2_ precipitate was subsequently lyophilized and then subjected to calcination at 350 °C for 2 h under a nitrogen (N_2_) atmosphere to yield NiO.

### 2.4. Preparation of CsPbI_3_

The synthesis of the CsPbI_3_ powders was accomplished through a chemical precipitation method using CsI and PbI_2_ as precursor materials, with methanol as the solvent [26]. First, 10 mL of methanol were used to dissolve 1.0 mM of CsI. After that, the solution was heated in a water bath to about 60 °C; 4 mL of n-butyl acetate were used to dissolve 1.0 mM of PbI_2_ powders in a different container. After carefully adding the PbI_2_ solution to the heated CsI solution, the mixture was thoroughly mixed. Fine yellow CsPbI_3_ powders precipitated as a result of this. To make sure the reaction was finished, the mixture was stirred for a further half hour at a speed of 300 revolutions per minute (rpm) in a water bath set at 60 °C. Centrifugation was used to separate and twice wash the solid CsPbI_3_ precipitates with n-butyl acetate. The cleaned CsPbI_3_ precipitates were then dried in an oven at 80 °C overnight to obtain the desired CsPbI_3_ powders.

### 2.5. Preparation of Ti_3_C_2_-MXene/NiO/CsPbI_3_ Composites

The Ti_3_C_2_-MXene/NiO/CsPbI_3_ composites were prepared via a hydrothermal treatment using an aqueous solution containing Ti_3_C_2_-MXene nanosheets, NiO, and CsPbI_3_ powders. Initially, 50 mg of Ti_3_C_2_-MXene nanosheets were dissolved in 50 mL of deionized (DI) water. To achieve delamination, the mixture was subjected to stirring and probe sonication for 5–10 min, respectively, at room temperature. This resulted in the formation of a homogeneous colloidal solution of Ti_3_C_2_-MXene nanosheets. Subsequently, 15 mg of NiO and 15 mg of CsPbI_3_ were added to the prepared colloidal solution. Again, the mixture was stirred and probe-sonicated for a duration of 5–10 min. The hydrothermal reaction was then started after the resultant solution was put into a 100 mL stainless steel container lined with Teflon. For twelve hours, the reaction was run at 160 °C with a 2 °C per minute heat ramp-up rate. Following the completion of the hydrothermal reaction, room temperature was allowed to settle in the Teflon container. Centrifugation was used to gather the product, and the centrifuge ran for 5 min at 2000 RCF. After collecting the material, it was cleaned and then freeze-dried for 48 h to produce the final composite powder.

### 2.6. Characterizations

FT-IR spectra were collected for the Ti_3_C_2_-MXene nanosheets, NiO, Ti_3_C_2_-MXene/NiO, CsPbI_3_, and Ti_3_C_2_-MXene/NiO/CsPbI_3_ composites using a Nicolet 370 FTIR spectrometer (Waltham, MA, USA) with KBr disks. The XRD patterns of these materials were obtained using a Bruker AXS D8 DISCOVER diffractometer (Bruker, MA, USA) with Cu Ka (0.1542) radiation, spanning a 2θ range of 5 to 80°. Transmission electron microscopy (TEM) analysis was conducted with a JEM 2010 instrument (Tokyo, Japan). In the TEM analysis, the materials, including Ti_3_C_2_-MXene nanosheets, Ti_3_C_2_-MXene/NiO, and Ti_3_C_2_-MXene/NiO/CsPbI_3_ composites, were dispersed in ethanol using an ultrasonicator and subsequently deposited on a copper grid. Field emission-scanning electron microscopy (FE-SEM) was utilized for examining the morphology of these materials and was performed using a Zeiss SUPRA 55 instrument (Oberkochen, Germany). The elemental composition of the Ti_3_C_2_-MXene/NiO/CsPbI_3_ composites was investigated using X-ray photoelectron spectroscopy (XPS, Thermo ESCALAB 250, Waltham, MA, USA). The optical properties of the Ti_3_C_2_-MXene nanosheets, Ti_3_C_2_-MXene/NiO, and Ti_3_C_2_-MXene/NiO/CsPbI_3_ composites were characterized through UV–visible spectroscopy (UV–Vis) using a Hitachi U-3010 instrument (Tokyo, Japan). Fluorescence spectral studies were performed with a Cary Eclipse fluorescence spectrometer or Edinburgh Instrument, UK FLS 900 luminescence spectrometer (Livingston, MN, USA). Under simulated UV light, an electrochemical workstation (Metrohm Autolab M204 multichannel potentiostat galvanostat with Nova 2.1.4 software) was used to measure electrochemical impedance spectra (EIS) in the frequency range of 10^5^ to 0.01 Hz. The reference electrode and counter electrodes in the three-electrode system were Ag/AgCl and Pt, respectively.

### 2.7. Photocatalytic Degradation of Dye

The assessment of the newly synthesized photocatalyst performance in crystal-violet (CV) degradation under UV-light irradiation. It involved dispersing a set quantity of the photocatalysts (25 mg) in 100 mL of CV-dye solutions with a concentration of 15 mg/L. A UV protection cabinet equipped with a UV medium-pressure immersion lamp was used during the experiments. The photocatalyst, composed of Ti_3_C_2_-MXene nanosheets, Ti_3_C_2_-MXene/NiO, and Ti_3_C_2_-MXene/NiO/CsPbI_3_ composites was mixed with CV and homogenized using a magnetic stirrer. To validate the equilibrium adsorption–desorption characteristics, a UV–Vis measurement was conducted for 30 min in a dark space before light illumination. A 400 W lamp producing a line spectrum in the ultraviolet and visible range (200–800 nm) and a high-power output density of about 100 W/cm^2^ in the UVC range (200–300 nm) served as the UV-light source. Subsequently, the light source was activated to initiate the photocatalytic dye degradation process. At specific time intervals (every 15 min), 3 mL aliquots were extracted, and these aliquots were then centrifuged to separate the nanosized photocatalyst during the degradation process. Photocatalytic degradation tests were conducted three times, and the average values were reported. The CV concentrations were measured using a UV–Vis spectrometer, and the removal efficiency (%) was determined using the following formula.

Removal efficiency (%) = (C_0_ − C_t_)/C_0_ × 100%

where C_0_ and C_t_ (mg/L) are the initial and final concentrations of CV at time t, respectively.

To assess the stability and recyclability of the Ti_3_C_2_-MXene/NiO/CsPbI_3_ composites, a series of recycling photocatalytic experiments were carried out. These experiments consisted of three successive cycles for the degradation of CV. After each photocatalytic cycle, the Ti_3_C_2_-MXene/NiO/CsPbI_3_ composites were collected, subjected to centrifugation, and washed multiple times with double-distilled water. Subsequently, they were dried at a temperature of 60 °C. After that, the recovered photocatalyst was used again in the same conditions as the first experiment to break down CV dye.

## 3. Results and Discussion

### 3.1. Surface Analysis of Ti_3_C_2_-MXene/NiO/CsPbI_3_ Composites

**Figure 1:**
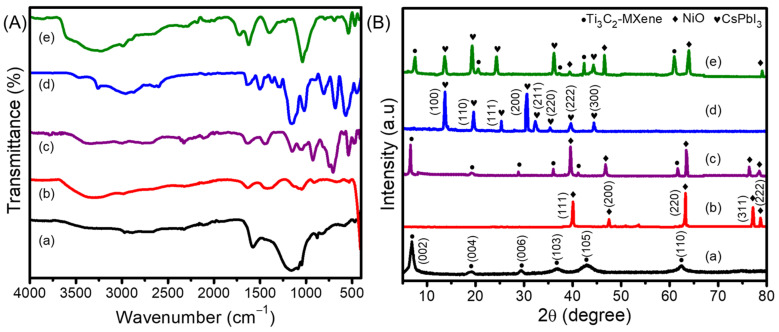
(**A**) FTIR spectra and (**B**) XRD pattern of (**a**) Ti_3_C_2_-MXene nanosheets, (**b**) NiO, (**c**) Ti_3_C_2_-MXene/NiO, (**d**) CsPbI_3_, and (**e**) Ti_3_C_2_-MXene/NiO/CsPbI_3_ composites.

The chemical functional groups present in the as-prepared materials, including Ti_3_C_2_-MXene nanosheets, NiO, Ti_3_C_2_-MXene/NiO, CsPbI_3_, and the Ti_3_C_2_-MXene/NiO/CsPbI_3_ composites, were characterized through the FTIR spectra, as illustrated in Figure 1A.

In the FTIR spectra, the Ti_3_C_2_-MXene nanosheets exhibited distinct bands at 3428, 1629, 1388, 1096, and 655 cm^−1^. These bands corresponded to the stretching vibrations of –OH, C=O, O–H, C–F, and Ti–O bonds, respectively. These observations were consistent with a previously reported study [27]. The FTIR spectrum of NiO revealed broad absorption bands at 3395 and 1381 cm^−1^ associated with O–H stretching vibrations. Additional stretching vibrations were observed at 1629 and 1021 cm^−1^, which were attributed to the surface-adsorbed moisture and physical absorption of CO_2_ during sample preparation. A distinctive absorption band at 459 cm^−1^ supported the formation of NiO nanoparticles. The FTIR spectrum of the Ti_3_C_2_-MXene/NiO displayed broadbands at 3451 and 1598 cm^−1^, supporting the presence of a hydroxyl functional group (O–H) on the surface of Ti_3_C_2_-MXene/NiO. The characteristic absorption band of NiO was evident at 462 cm^−1^, corresponding to the tensile vibration of Ni–O [28]. The antisymmetric C–H stretching modes were identified in the FTIR spectrum of CsPbI_3_ by the signals at 2921 and 2849 cm^−1^, and the COO– stretching modes were identified in the signals at 1531 and 1411 cm^−1^ [29]. Furthermore, the FTIR spectra displayed peaks at 569, 1028, 1112, 1181, 1381, and 1628 cm^−1^, corresponding to –C–I, –CH, –NH, –C=O, –C=C, and –OH bonds, respectively. These peaks aligned with the characteristic bonds of both CsPbI_3_ and Ti_3_C_2_-MXene/NiO, indicating the presence of an interfacial interaction between CsPbI_3_ and Ti_3_C_2_-MXene/NiO. In the Ti_3_C_2_-MXene/NiO/CsPbI_3_ composites, most of these peaks were present with slightly reduced intensities compared to Ti_3_C_2_-MXene/NiO and CsPbI_3_ alone. However, the characteristic peaks of CsPbI_3_ and Ti_3_C_2_-MXene/NiO in the Ti_3_C_2_-MXene/NiO/CsPbI_3_ composites exhibited significantly higher intensities than in Ti_3_C_2_-MXene/NiO alone. This observation indicated a stronger interaction effect between CsPbI_3_ and the few-layered Ti_3_C_2_-MXene/NiO, which is consistent with the XRD results.

To investigate the crystallographic details and phase composition of the materials, XRD patterns for Ti_3_C_2_-MXene nanosheets, NiO, Ti_3_C_2_-MXene/NiO, CsPbI_3_, and Ti_3_C_2_-MXene/NiO/CsPbI_3_ composites were obtained and are presented in Figure 1B. For the Ti_3_C_2_-MXene nanosheets, distinct peaks were observed at 2θ values of 7.1°, 19.3°, 28.9°, 36.9°, 42.4°, and 62.5°. These peaks corresponded to the (002), (004), (006), (103), (105), and (110) lattice planes of MXene, respectively [30]. In the case of NiO, the characteristic peaks appeared at 39.2°, 47.5°, 63.2°, 76.3°, and 78.4°, which could be assigned to the (111), (200), (220), (311), and (222) lattice planes of NiO, respectively. As for the Ti_3_C_2_-MXene/NiO composite, its XRD pattern displayed characteristic peaks corresponding to both Ti_3_C_2_-MXene and NiO, confirming the successful synthesis of the composite [31]. Notably, the (002) peak of the Ti_3_C_2_-MXene/NiO composite shifted to a lower angle compared to pristine Ti_3_C_2_-MXene, indicating the intercalation of NiO between the interlayers of Ti_3_C_2_-MXene. The XRD pattern of nanocrystalline CsPbI_3_ revealed peaks at 2θ values of 13.7°, 19.6°, 25.2°, 29.7°, 32.3°, 35.6°, 39.7°, and 44.8°, corresponding to (100), (110), (111), (200), (211), (220), (222), and (300) lattice planes, respectively. These sharp diffraction peaks demonstrated the excellent crystallinity of the CsPbI_3_ [32]. The Ti_3_C_2_-MXene/NiO/CsPbI_3_ composites exhibited nearly all the specific peaks of Ti_3_C_2_-MXene, NiO, and CsPbI_3_, confirming the successful synthesis of the composite. Interestingly, the typical XRD peaks of Ti_3_C_2_-MXene (29.6°), NiO (78.2°), and CsPbI_3_ (32.3° and 34.6°) were not detected. This absence could be attributed to the full coverage of NiO on CsPbI_3_ or the presence of NiO and CsPbI_3_ crystals on the surface of Ti_3_C_2_-MXene, resulting in a modified diffraction pattern.

### 3.2. Morphological Properties of Ti_3_C_2_-MXene/NiO/CsPbI_3_ Composites

**Figure 2:**
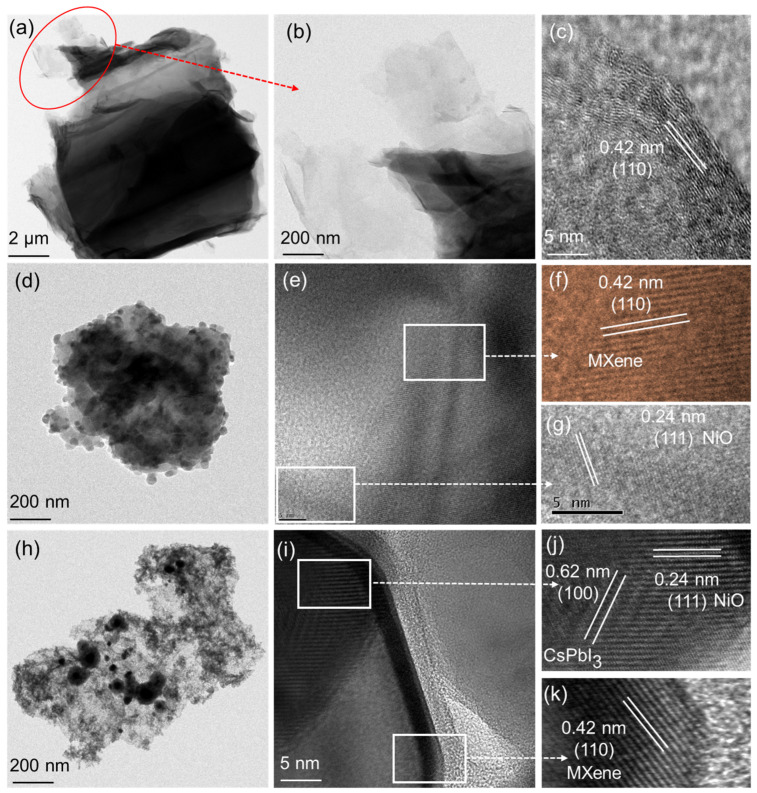
TEM images of (**a**,**b**) Ti_3_C_2_-MXene nanosheets and (**c**) the corresponding HRTEM image, TEM images of (**d**) Ti_3_C_2_-MXene/NiO and (**e**–**g**) the corresponding HRTEM image, and TEM images of (**h**) Ti_3_C_2_-MXene/NiO/CsPbI_3_ composites and (**i**–**k**) the corresponding HRTEM image.

The morphology of the as-prepared Ti_3_C_2_-MXene nanosheets, Ti_3_C_2_-MXene/NiO, and Ti_3_C_2_-MXene/NiO/CsPbI_3_ composites were analyzed by transmission electron microscopy (TEM). Figure 2a,b provide TEM images of the Ti_3_C_2_-MXene nanosheets, confirming their thin and electron-transparent nature, with a thickness comparable to graphene. Some local regions exhibit folding, which is attributed to their high flexibility and elasticity [33]. Figure 2c presents a high-resolution TEM image of the Ti_3_C_2_-MXene, reaffirming their graphene-like morphology. The lattice fringes observed in this image, with a spacing of 0.42 nm, correspond to the typical (110) plane of the layered structure of Ti_3_C_2_-MXene nanosheets. Moving on to the Ti_3_C_2_-MXene/NiO composite, the TEM images in Figure 2d clearly illustrate the well-dispersed NiO particles on the thin and transparent Ti_3_C_2_-MXene nanosheets without altering the initial structure of the Ti_3_C_2_-MXene nanosheets [34]. Differentiable lattice fringes and grain boundaries between the NiO particles and Ti_3_C_2_-MXene nanosheets can be seen in the high-resolution TEM (HRTEM) image displayed in Figure 2e–g. The image-derived lattice spacing of 0.24 nm is consistent with the cubic NiO (111) crystal-plane spacing. A TEM analysis also confirms that the lattice spacing of 0.42 nm corresponds to the (110) crystal plane of Ti_3_C_2_-MXene [25]. An additional TEM analysis (Figure 2h) demonstrates the presence of CsPbI_3_ particles loaded onto the Ti_3_C_2_-MXene/NiO composite. Figure 2h reveals that NiO and CsPbI_3_ particles, each of a narrow size, entirely cover the surface of Ti_3_C_2_-MXene. Furthermore, the HRTEM image of the Ti_3_C_2_-MXene/NiO/CsPbI_3_ composites reveals distinctly different phases [35]. The HRTEM lattice fringe pattern displays interplanar spacings of 0.62, 0.24, and 0.42 nm (Figure 2i–k), corresponding to the (100) plane of CsPbI_3_, the (111) plane of NiO, and the (110) plane of Ti_3_C_2_-MXene nanosheets, respectively. These findings are in good agreement with the XRD results.

**Figure 3:**
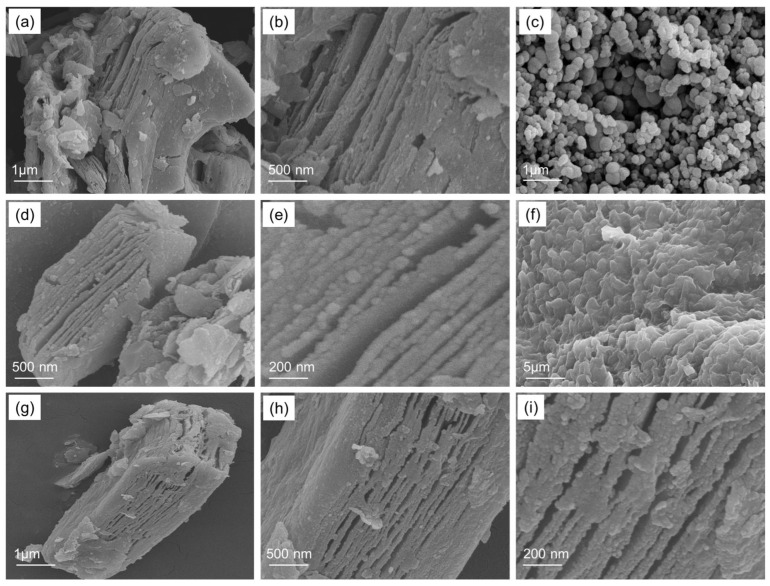
FE-SEM images of (**a**,**b**) Ti_3_C_2_-MXene nanosheets, (**c**) NiO, (**d**,**e**) Ti_3_C_2_-MXene/NiO, (**f**) CsPbI3, and (**g**–**i**) Ti_3_C_2_-MXene/NiO/CsPbI_3_ composites.

Figure 3 represents the FESEM image of the Ti_3_C_2_-MXene nanosheets, NiO, Ti_3_C_2_-MXene/NiO, CsPbI_3_, and Ti_3_C_2_-MXene/NiO/CsPbI_3_ composites phase. In Figure 3a,b, the SEM micrographs illustrate the distinctive structure of Ti_3_C_2_-MXene nanosheets, characterized by a typical 2D and sheet-like arrangement with only a few layers, resembling the graphene-like structure [25]. The surface of these nanosheets appears smooth, and the presence of a well-defined layered structure confirms the existence of Ti_3_C_2_-MXene nanosheets (Figure 3b). Figure 3c presents SEM micrographs of the NiO nanoparticles, displaying a generally spherical shape with some degree of aggregation. In contrast, Figure 3d,e show SEM images of the Ti_3_C_2_-MXene/NiO composite. After combining with NiO, the surface of Ti_3_C_2_-MXene/NiO becomes notably rough, with some NiO particles covering the surface of Ti_3_C_2_-MXene, while others are randomly embedded within the layers of Ti_3_C_2_-MXene [25,28]. For the SEM images of CsPbI_3_ shown in Figure 3f, the majority of the particles exhibit a spherical shape, with a few particles exhibiting faceted (cubic) shapes and a brighter contrast [36]. In Figure 3g–i, typical SEM images of the Ti_3_C_2_-MXene/NiO/CsPbI_3_ composites reveal that Ti_3_C_2_-MXene nanosheets are evenly covered with spherical NiO and CsPbI_3_ particles, while the ordered layer structure of Ti_3_C_2_-MXene is still retained. These morphologies confirm the successful seeding and growth of NiO and CsPbI_3_ particles on the surface of Ti_3_C_2_-MXene, resulting in an enhanced surface roughness [25].

**Figure 4:**
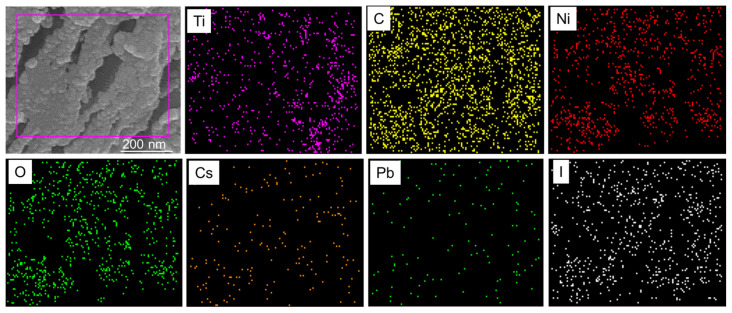
FE-SEM image and the corresponding EDS elemental mapping of Ti_3_C_2_-MXene/NiO/CsPbI_3_ composites.

To further corroborate the formation of the Ti_3_C_2_-MXene/NiO/CsPbI_3_ composites, elemental mapping was performed using energy-dispersive spectroscopy (EDS) (Figure 4). As depicted in Figure 4, NiO particles envelop CsPbI_3_, and this combined structure is adhered to the Ti_3_C_2_-MXene nanosheets. Elemental mapping reveals the distribution of various elements, such as Ti, C, Ni, O, Cs, Pb, and I, within the nanocomposite structure, providing further evidence of composite formation [25,37].

### 3.3. Elemental Composition of Ti_3_C_2_-MXene/NiO/CsPbI_3_ Composites

**Figure 5:**
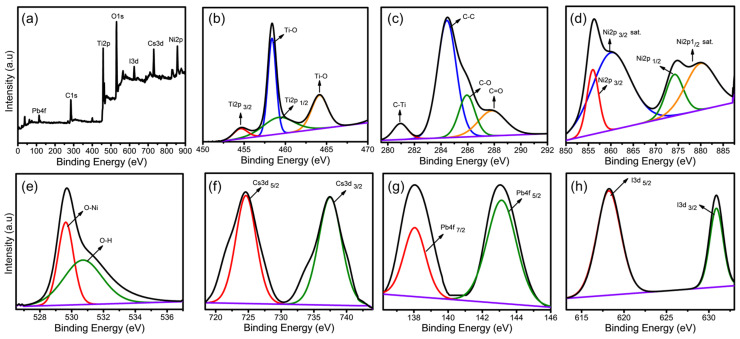
(**a**) XPS survey scan spectrum of Ti_3_C_2_-MXene/NiO and high-resolution XPS spectra of elements (**b**) Ti2p, (**c**) C1s, (**d**) Ni2p, (**e**) O1s, (**f**) Cs3d, (**g**) Pb4f, and (**h**) I3d states for Ti_3_C_2_-MXene/NiO. (Black line; fitting and violet line; background).

X-ray photoelectron spectroscopy (XPS) analysis was performed to investigate the chemical composition of the Ti_3_C_2_-MXene/NiO/CsPbI_3_ composites. In Figure 5a, the XPS survey spectrum of Ti_3_C_2_-MXene/NiO/CsPbI_3_ composites revealed seven distinct peaks at various binding energies: 458.0 eV for Ti2p, 274.3 eV for C1s, 857.5 eV for Ni2p, 530.4 eV for O1s, 730.4 eV for Cs3d, 114.1 eV for Pb4f, and 625.5 eV for I3d [28,34]. Figure 5b illustrates the core-level spectrum of the Ti2p signal, which consists of two peaks (Ti2p_3/2_ and Ti2p_1/2_) with corresponding binding energies of 454.5 and 459.6 eV. A spin-energy separation of 5.1 eV was observed between Ti2p_3/2_ (454.5 eV) and Ti2p_1/2_ (459.6 eV) [28]. The C1s spectrum (Figure 5c) displays peaks at 280.9 eV, 284.3 eV, 285.9 eV, and 287.7 eV, which correspond to the characteristic bonds of C–Ti, C–C, C–O, and C=O, respectively. In Figure 5d, the core-level spectrum of Ni2p shows two noticeable satellite peaks (indicated as “Sat”) Ni2p_3/2_ and Ni2p_1/2_ at 860.2 and 879.8 eV, respectively. The peaks at 855.8 and 874.2 eV were attributed to the oxidation state of Ni2p_3/2_ and Ni2p_1/2_ [34]. Additionally, in the O1s XPS spectrum (Figure 5e), two distinct oxygen environments of NiO were observed. The O1s peak at 529.6 eV was attributed to O–Ni, while the peak at 530.7 eV was associated with O–H, confirming the presence of hydroxyl groups (Ti–OH) on the Ti_3_C_2_-MXene surface [28,34]. Furthermore, the Cs3d XPS spectrum displayed two symmetric peaks at binding energies of 737.3 and 724.2 eV with a separation of 13.1 eV, corresponding to the Cs3d3/2 and 3d5/2 energy levels, respectively (Figure 5f). The spectrum of Pb4f exhibited peaks at 4f_5/2_ (143.1 eV) and 4f_7/2_ (138.3 eV) with a separation of 4.8 eV, indicating the presence of Pb^2+^ ions (Figure 5g). In Figure 5h, the double peaks in the I3d spectrum, I3d_3/2_ (630.4 eV) and 3d_5/2_ (618.3 eV), were attributed to I^−^ [38]. The above results confirm the successful synthesis of CsPbI_3_ anchored on Ti_3_C_2_-MXene/NiO composites.

### 3.4. Optical Properties of Ti_3_C_2_-MXene/NiO/CsPbI_3_ Composites

**Figure 6:**
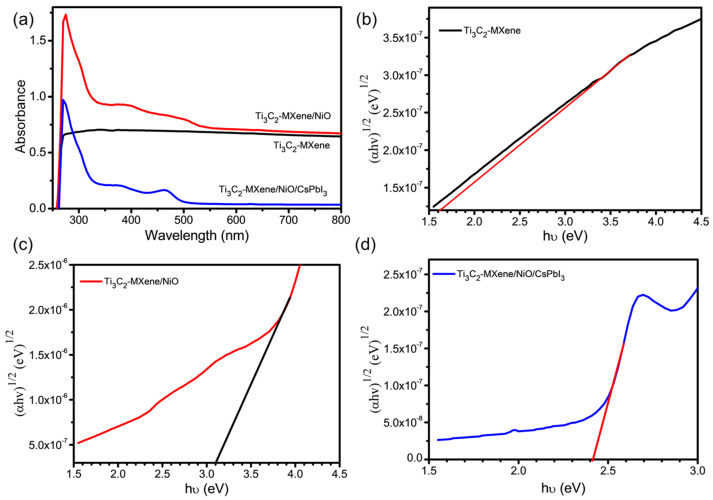
(**a**) UV–Visible absorption spectra of Ti_3_C_2_-MXene nanosheets, Ti_3_C_2_-MXene/NiO, and Ti_3_C_2_-MXene/NiO/CsPbI_3_ composites and Tauc plot of (**b**) Ti_3_C_2_-MXene nanosheets, (**c**) Ti_3_C_2_-MXene/NiO, and (**d**) Ti_3_C_2_-MXene/NiO/CsPbI_3_ composites from UV–Visible absorption spectroscopy.

The UV–vis absorption properties and band gap of the Ti_3_C_2_-MXene nanosheets, Ti_3_C_2_-MXene/NiO, and Ti_3_C_2_-MXene/NiO/CsPbI_3_ composites were investigated by UV–vis spectroscopy, as shown in Figure 6. Figure 6a presents the UV–Vis absorption spectrum of the Ti_3_C_2_-MXene nanosheets in a dilute aqueous medium, displaying distinct peaks at 275 nm [39]. When UV–Vis spectroscopy was performed on a mixed solution of Ti_3_C_2_-MXene/NiO, it is evident that the typical peak position of NiO was at 380 nm, and that of Ti_3_C_2_-MXene/NiO was also located at 380 nm [40]. Notably, a new absorption band appears at the position of a peak around 465 nm after the incorporation of CsPbI_3_. With successive additions of CsPbI_3_, the absorption spectra of the Ti_3_C_2_-MXene/NiO/CsPbI_3_ composites retained the characteristics of the native Ti_3_C_2_-MXene/NiO behavior. The peak position of NiO at 380 nm remained virtually unchanged, indicating that the structure of Ti_3_C_2_-MXene/NiO was unaffected by the addition of CsPbI_3_. However, the peaks of Ti_3_C_2_-MXene nanosheets shifted from 275 nm to 270 nm, confirming the successful addition of CsPbI_3_ to the Ti_3_C_2_-MXene/NiO composites. Additionally, the presented Figure 6b–d illustrates the determination of the energy band gap using the Tauc plots of Ti_3_C_2_-MXene nanosheets, Ti_3_C_2_-MXene/NiO, and Ti_3_C_2_-MXene/NiO/CsPbI_3_ composites. *αhυ* = A(*hυ* − E_g_)*^n^*; in this relation, *α* represents the absorption coefficient, *hυ* corresponds to the photon energy, E_g_ is the band gap, and *n* is set to 1/2 for direct transitions. Figure 6b–d features a plot of (*αhυ*)^2^ against the *hυ* axis for the determination of the band gap. The determined band gap values are 1.60 eV for the Ti_3_C_2_-MXene nanosheets and 3.10 eV for Ti_3_C_2_-MXene/NiO. Notably, the band gap of the Ti_3_C_2_-MXene/NiO/CsPbI_3_ composites experiences a slight reduction to 2.41 eV. This decrease in the band gap implies electronic interaction and enhanced coupling among the Ti_3_C_2_-MXene nanosheets, NiO, and CsPbI_3_ particles, resulting in modified optical properties [41].

### 3.5. Charge-Transfer Behavior of Ti_3_C_2_-MXene/NiO/CsPbI_3_ Composites

**Figure 7:**
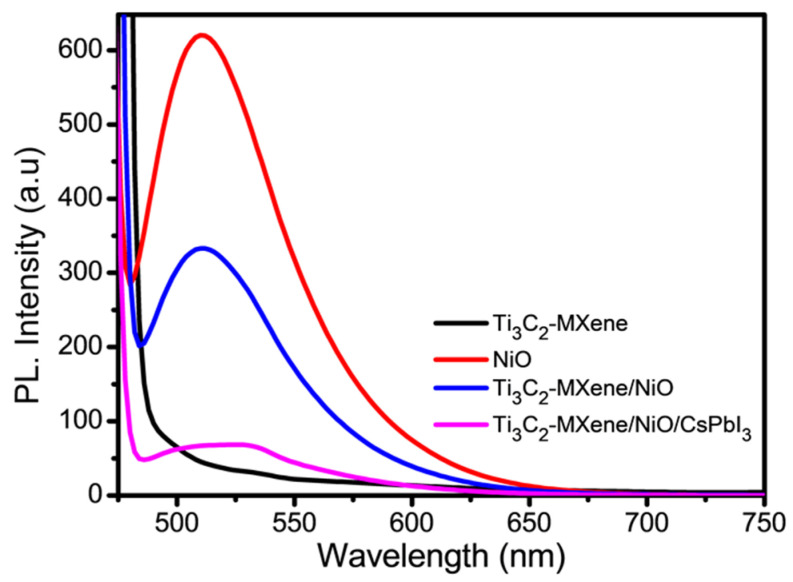
PL spectra of Ti_3_C_2_-MXene nanosheets, NiO, Ti_3_C_2_-MXene/NiO, and Ti_3_C_2_-MXene/NiO/CsPbI_3_ composites.

Photoluminescence (PL) spectroscopy was employed to examine the rate of recombination of photoinduced charge carriers in the prepared materials, as depicted in Figure 7.

The PL intensity serves as a measure of the semiconductor electronic behavior, particularly with regard to the charge recombination rate, which has a direct impact on the photocatalytic performance of the materials. The PL spectra of Ti_3_C_2_-MXene nanosheets, NiO, Ti_3_C_2_-MXene/NiO, and Ti_3_C_2_-MXene/NiO/CsPbI_3_ composites reveal emission peaks at 530, 517, 515, and 532 nm, respectively. The charge recombination rate is indicated by the peak’s intensity in these PL spectra. Consequently, in comparison to the Ti_3_C_2_-MXene/NiO/CsPbI_3_ composites, which show a weaker intensity peak, the PL spectra of Ti_3_C_2_-MXene/NiO, with a high peak intensity, indicate a higher electron–hole recombination rate [41]. Because of this, the process of creating Ti_3_C_2_-MXene/NiO/CsPbI_3_ composites decreases the amount of photogenerated charge carriers that recombine, which increases the quantity of charge carriers that are available for photocatalytic degradation [42].

**Figure 8:**
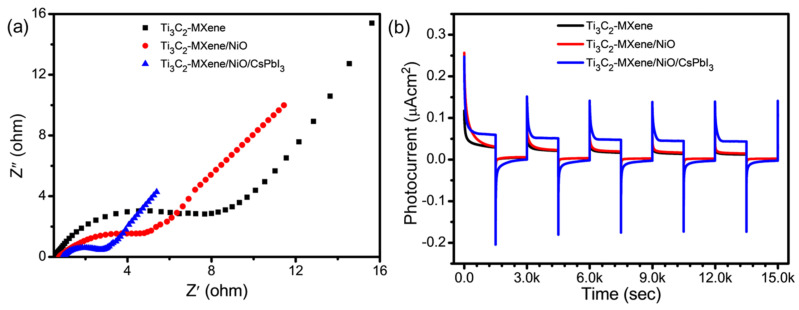
(**a**) Electrochemical impedance spectra and (**b**) photocurrent response of Ti_3_C_2_-MXene nanosheets, Ti_3_C_2_-MXene/NiO, and Ti_3_C_2_-MXene/NiO/CsPbI_3_ composites.

In the study, to gain insights into the relationship between the electrochemical performance and the resistance behavior of various materials, including Ti_3_C_2_-MXene nanosheets, Ti_3_C_2_-MXene/NiO, and Ti_3_C_2_-MXene/NiO/CsPbI_3_ composites, they used Nyquist plots derived from electrochemical impedance spectroscopy (EIS) tests, as depicted in Figure 8a. Nyquist plots are powerful tools for analyzing electrochemical systems. In these plots, the impedance spectra are represented by characteristic shapes. In the high-frequency regions, one can observe sharp semicircles that intersect the real axis, whereas, in the low-frequency regions, nearly vertical lines become apparent. These shapes provide valuable information about the material electrical properties. The Ti_3_C_2_-MXene nanosheets exhibited a distinctive behavior in the Nyquist plot, with their curve and the horizontal axis intersecting at the smallest point in Figure 8a. This intersection indicates that this particular sample has the lowest equivalent internal resistance among all the tested materials. Notably, the Ti_3_C_2_-MXene nanosheets lack a significant arc in the high-frequency region, implying low charge-transfer resistance. This is a promising sign for their electrochemical performances. Conversely, the Ti_3_C_2_-MXene/NiO composite displayed a larger semicircular diameter in the high-frequency region, suggesting a higher internal resistance to electron conduction. This behavior can be attributed to the presence of NiO sheets on the surface of Ti_3_C_2_-MXene, which contribute to this increased resistance. The Ti_3_C_2_-MXene/NiO/CsPbI_3_ composites stood out in the Nyquist plot by displaying the smallest diameter among the samples [43]. This observation confirmed the lowest resistance and highest conductivity compared to the Ti_3_C_2_-MXene nanosheets and Ti_3_C_2_-MXene/NiO samples [44,45]. This enhanced interfacial charge transfer within the composites is a positive indication of their superior electrochemical performance, ultimately contributing to improved photocatalytic activity. Therefore, the Nyquist plots derived from EIS tests offer valuable insights into the electrical properties and performance of the tested materials, shedding light on their suitability for various applications, particularly in the context of photocatalysis. To further explore the efficacy of separating photoexcited electrons and holes, we conducted a series of photocurrent measurements on Ti_3_C_2_-MXene nanosheets, Ti_3_C_2_-MXene/NiO, and Ti_3_C_2_-MXene/NiO/CsPbI_3_ composite materials. The photocurrent profiles, presented in Figure 8b, illustrate the periodic on–off responses to UV light illumination for these materials. Notably, the Ti_3_C_2_-MXene/NiO/CsPbI_3_ composite exhibits a superior photocurrent response compared to the Ti_3_C_2_-MXene nanosheets and the Ti_3_C_2_-MXene/NiO, which is consistent with the observed photocatalytic activity. Measurements in a 3 mol L^−1^ KOH solution reveal a photocurrent density of 0.18 µA cm^−2^ for the Ti_3_C_2_-MXene/NiO/CsPbI_3_ composite superior to 0.15 µA cm^−2^ for Ti_3_C_2_-MXene nanosheets and 0.16 µA cm^−2^ for Ti_3_C_2_-MXene/NiO. This outcome indicates that the integration of Ti_3_C_2_-MXene/NiO with CsPbI_3_ enhances photocurrent density, suggesting improved separation efficiency of photoexcited carriers. Therefore, these findings highlight the robust capacity of the Ti_3_C_2_-MXene/NiO/CsPbI_3_ composite to transfer and generate photoexcited charge carriers under UV light, an essential factor in supplementing its photocatalytic performance.

### 3.6. Photocatalytic Degradation of Dye

**Figure 9:**
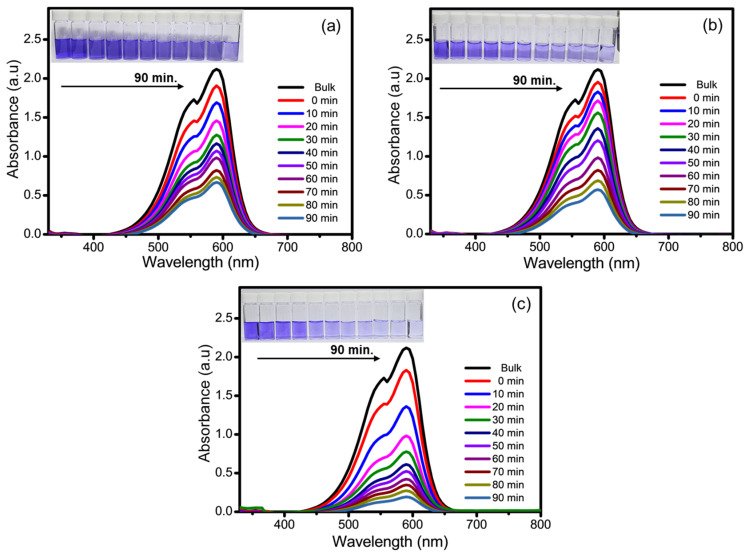
Absorption spectra of CV-dye photodegradation over, (**a**) Ti_3_C_2_-MXene nanosheets, (**b**) Ti_3_C_2_-MXene/NiO, and (**c**) Ti_3_C_2_-MXene/NiO/CsPbI_3_ composites.

The photocatalytic performance of the Ti_3_C_2_-MXene nanosheets, Ti_3_C_2_-MXene/NiO, and Ti_3_C_2_-MXene/NiO/CsPbI_3_ composites was assessed by the degradation of crystal-violet (CV) aqueous solution under UV-light irradiation. During the initial hour of the investigation, the degradation process commenced in dark conditions and continued for an additional 1.5 h with the presence of UV-light irradiation. In this study, the photocatalytic degradation of crystal violet (CV) was monitored at 590 nm, since there was no significant shift in the main peak of CV during the photocatalytic experiment [46]. The recorded UV–visible absorption spectra of the CV dye are illustrated in Figure 9a–c.

**Figure 10:**
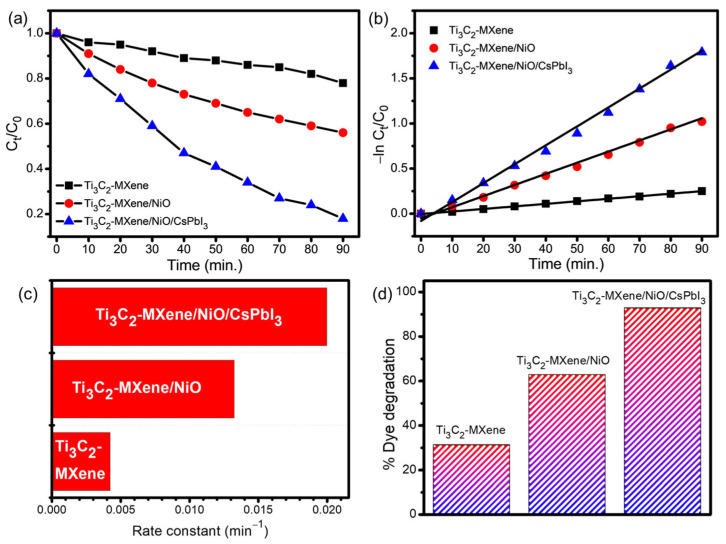
Degradation curves profiles of CV dye over Ti_3_C_2_-MXene nanosheets, Ti_3_C_2_-MXene/NiO, and Ti_3_C_2_-MXene/NiO/CsPbI_3_ composites, (**a**) the degradation rate, (**b**) linear kinetic, (**c**) rate constant, and (**d**) percentage degradation of dye.

Photocatalytic degradation of CV by the Ti_3_C_2_-MXene-based composite of NiO and CsPbI_3_ demonstrated excellent performance, attributed to the remarkable properties of Ti_3_C_2_-MXene, including its large surface area and surface functional groups. As the UV-light exposure duration increased, the color of the solutions containing Ti_3_C_2_-MXene nanosheets, Ti_3_C_2_-MXene/NiO, and Ti_3_C_2_-MXene/NiO/CsPbI_3_ composites also changed. The results for photodegradation are presented in the inset of Figure 9a–c. The degradation efficiency of the Ti_3_C_2_-MXene nanosheets, Ti_3_C_2_-MXene/NiO, and Ti_3_C_2_-MXene/NiO/CsPbI_3_ composites can be explained in terms of C_t_/C_0_, where C_0_ represents the initial concentration of the CV dye and C_t_ represents the concentration at a specific time interval (Figure 10a) [47]. The photocatalytic performance of these materials was evaluated based on the kinetics of CV-dye photodegradation, and it was found to follow first-order kinetics [48,49]. A straight line is obtained in Figure 10b when plotting −ln(C_t_/C_0_) against time (t), and the rate constant ‘k’ in min^−1^ can be calculated from the slope of this straight line [50]. The rate constant (k) values, as shown in Figure 10c, for the degradation of CV dye by Ti_3_C_2_-MXene nanosheets, Ti_3_C_2_-MXene/NiO, and Ti_3_C_2_-MXene/NiO/CsPbI_3_ composites are 0.0042, 0.0132, and 0.0199 min^−1^, respectively. The faster degradation of CV dye with Ti_3_C_2_-MXene/NiO/CsPbI_3_ composites is primarily attributed to the effective charge separation within these composites, possibly resulting from the formation of a heterojunction between Ti_3_C_2_-MXen, NiO, and CsPbI_3_ [51]. In addition, the Ti_3_C_2_-MXene nanocomposite contains NiO and CsPbI_3_, which not only provide a large surface area to improve CV-dye adsorption but also suppress electron and hole recombination, enhancing photocatalytic activity [48,49,50,51,52].

The UV–visible profiles clearly show that Ti_3_C_2_-MXene/NiO/CsPbI_3_ composites degrade CV dye faster compared to Ti_3_C_2_-MXene nanosheets and Ti_3_C_2_-MXene/NiO. The percentage of CV-dye degradation was recorded as 31.3%, 62.9%, and 92.8% for Ti_3_C_2_-MXene nanosheets, Ti_3_C_2_-MXene/NiO, and Ti_3_C_2_-MXene/NiO/CsPbI_3_ composites, respectively (Figure 10d).

The enhanced catalytic efficiency of Ti_3_C_2_-MXene/NiO (62.9%) compared to Ti_3_C_2_-MXene nanosheets (31.3%) is attributed to the defects generated by doping. Furthermore, the dopants act as trapping agents for holes and electrons, reducing their recombination phenomenon [53]. In the case of Ti_3_C_2_-MXene/NiO/CsPbI_3_ composites, the percentage of CV-dye degradation reaches 92.8%. This remarkable performance can be attributed to the Ti_3_C_2_-MXene nanosheets, which prevent the agglomeration of NiO and CsPbI_3_, resulting in an increased surface-to-volume ratio. Consequently, Ti_3_C_2_-MXene/NiO/CsPbI_3_ composites, with their larger surface area, adsorb a higher amount of CV dye, leading to a promising catalytic efficiency of 92.8%.

**Figure 11:**
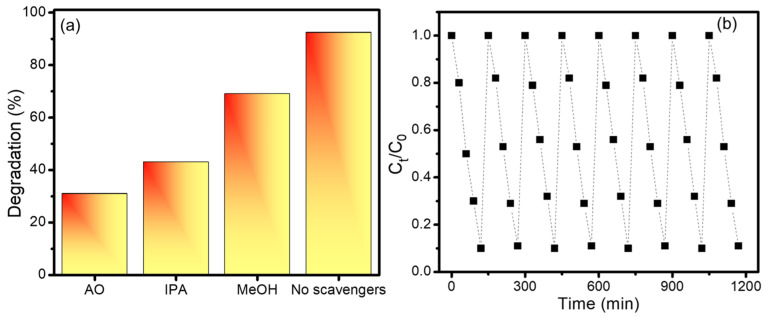
(**a**) Effect of different scavengers and (**b**) cyclic stability performance of Ti_3_C_2_-MXene/NiO/CsPbI_3_ composites in the photocatalytic removal of CV.

Radical scavenger experiments were carried out for the photocatalytic decomposition of CV in the presence of different scavengers in order to investigate the photocatalytic reaction mechanism of Ti_3_C_2_-MXeneNiO/CsPbI_3_ composites towards CV [54]. Using methanol (MeOH), isopropanol (IPA), and ammonium oxalate (AO) as scavengers, Figure 11a shows the photocatalytic breakdown of CV and validates the effects of superoxide radicals (•O_2_^−^), hydroxyl radicals (•OH), and photogenerated holes (h^+^), respectively. The substantial contribution of photogenerated holes to CV breakdown is confirmed by the noticeably lower photodegradation of CV (31.2%) in the presence of AO. Nonetheless, the moderate decrease in photodegradation (43.4% and 69.2%) when IPA and MeOH are present validates the partial contribution of hydroxyl radicals (•OH) [55]. Therefore, the photodegradation of CV is primarily mediated by hydroxyl radicals (•OH) and photogenerated holes (h^+^) [56]. Additionally, the reusability of Ti_3_C_2_-MXeneNiO/CsPbI_3_ composites was explored through eight successive cycles of CV degradation, as illustrated in Figure 11b.

It is noteworthy that a minimal decline of 2.9% (from 92.3% to 89.4%) in the photocatalytic degradation activity of CV dye was observed. This slight reduction might be attributed to the potential loss of catalyst material occurring during the centrifugation, drying, and washing steps involved in the recovery process. This underscores the importance of considering the various stages of the experimental procedure and their potential impact on the composite performance over multiple cycles.

### 3.7. Proposed Photocatalytic Mechanism

**Figure 12:**
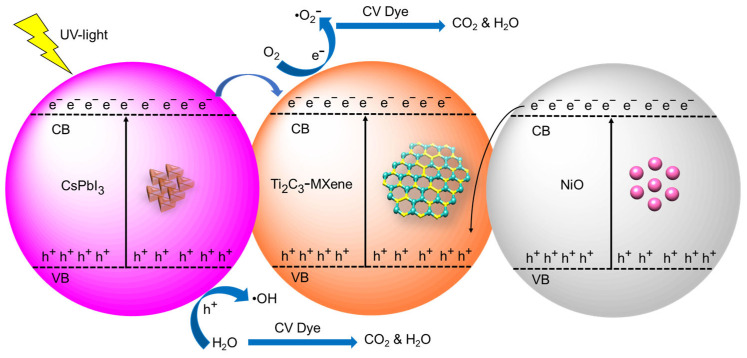
Schematic illustration of the proposed mechanism for photocatalytic degradation of CV dye using Ti_3_C_2_-MXene/NiO/CsPbI_3_ composites photocatalyst.

The efficiency of photoinduced carrier migration, transfer, and separation during the photocatalytic degradation process was examined using photoluminescence (PL) spectra [57]. PL emission usually results from photoexcited electron–hole pairs recombining [58]. A semiconductor with a lower PL intensity has a higher photocatalytic activity. Figure 12 shows a schematic representation of a suggested photocatalytic mechanism for the improved performance of Ti_3_C_2_-MXene/NiO/CsPbI_3_ composites based on the earlier findings.

In the PL spectra, Ti_3_C_2_-MXene nanosheets exhibit weak intensity, while NiO and Ti_3_C_2_-MXene/NiO exhibit the strongest PL intensity (Figure 7), indicating rapid recombination of photoexcited charge carriers [59]. The PL intensity of Ti_3_C_2_-MXene/NiO/CsPbI_3_ composites decreases following modification with CsPbI_3_ and NiO, indicating that CsPbI_3_ and NiO, functioning as an electron mediator, efficiently encourages the separation of photoexcited charge carriers. As a result, the composites Ti_3_C_2_-MXene/NiO/CsPbI_3_ show the lowest PL-emission intensity, suggesting a higher carrier-separation rate and greater production of reactive species for the degradation of pollutants. These PL spectroscopy findings are consistent with the evaluation of photocatalytic activity, confirming that photogenerated carrier-separation efficiency in semiconductors does, in fact, influence photocatalytic activity. In the previous literature, when exposed to light, MXenes generate electron–hole pairs, with electrons in the conduction band participating in reduction reactions and holes in the valence band engaging in oxidation reactions [60]. In the context of Cr(VI) reduction, for instance, MXenes facilitate the conversion of toxic Cr(VI) species to less harmful forms [61]. As a catalyst, MXenes remain unaltered during the reaction, providing surfaces for reactant adsorption and bringing them into proximity to enhance reaction possibility [62]. The final products of photocatalysis depend on the specific reactants, such as the reduction of Cr(VI) leading to Cr(III) species [63]. In addition, the surface properties, including defects and functional groups, are crucial for determining active sites, emphasizing the importance of understanding MXene surface chemistry to optimize the photocatalytic activity [64]. The photocatalytic systems achieving high solar-to-hydrogen efficiency in photocatalytic water splitting are crucial for renewable hydrogen production. Various materials and strategies are employed to enhance the efficiency of this process, including the use of semiconductor photocatalysts and optimizing reaction conditions [65]. ZnIn_2_S_4_ is a semiconductor material used in photocatalysis for hydrogen evolution. Designing heterostructured photocatalysts involves combining different materials to create interfaces that enhance charge separation and improve the overall efficiency in hydrogen-evolution reactions [66]. Also, the metal–organic frameworks (MOFs) are porous materials with unique properties. Constructing double Z-scheme heterojunctions involves creating specific arrangements of MOFs to facilitate efficient charge transfer and enhance photocatalytic activity, particularly in the degradation of pollutants [67]. This topic likely involves the development of a strategy to spatially separate redox reactions in order to achieve efficient and highly selective photoconversion of amines to imines [68]. Furthermore, this kind of research can have implications for the design of more precise and selective chemical processes. This proposed mechanism states that photogenerated electrons from the CsPbI_3_ conduction band (CB) migrate to Ti_3_C_2_-MXene and NiO CB migrate to the Ti_3_C_2_-MXene valence band (VB), resulting in Fermi levels that coincide [69]. These electrons then mix with the h^+^ produced by photocatalysis in the VB of Ti_3_C_2_-MXene/NiO VB, which may lead to a low migration rate or the accumulation of charge carriers. In order to mitigate these problems, CsPbI_3_ is incorporated at the interface between NiO and Ti_3_C_2_-MXene, acting as a bridge to accelerate the rate of electron migration from NiO to Ti_3_C_2_-MXene [70]. The electrons in the CB of Ti_3_C_2_-MXene react with trapped O_2_ to form •O_2_^−^ and h^+^ in the VB of CsPbI_3_ and NiO, which react with water molecules to form •OH radicals. The CV dye is then reacted with by these free radicals, producing CO_2_ and H_2_O [71]. This proposed mechanism illustrates how the introduction of NiO and CsPbI_3_ facilitates efficient charge separation and promotes the degradation of pollutants in the photocatalytic process. The reactions are as follows:

Ti_3_C_2_-MXene/NiO/CsPbI_3_ + hν → h^+^ + e^−^

e^−^ + O_2_ → •O_2_^−^

h^+^ + H_2_O → •OH + H^+^

e^−^ + CV → Reduction products

h^+^ + CV → Oxidation products

•OH + CV → Degradation products (CO_2_ + H_2_O)

•O_2_^−^ + CV→ Degradation products (CO_2_ + H_2_O)

## 4. Conclusions

A novel ternary composite composed of Ti_3_C_2_-MXene/NiO/CsPbI_3_ is successfully synthesized and studied as the best photocatalyst for the degradation of crystal-violet dye. The as-prepared Ti_3_C_2_-MXene/NiO/CsPbI_3_ composite was investigated in comparison with Ti_3_C_2_-MXene nanosheets, NiO, CsPbI_3_, and a binary composite of Ti_3_C_2_-MXene/NiO. Various properties of the composite were analyzed, including optical, structural, and morphological properties, using techniques such as FTIR, XRD, TEM, SEM–EDS mapping, XPS, UV–Vis, and PL spectroscopy. The study found that the Ti_3_C_2_-MXene/NiO/CsPbI_3_ composite exhibited superior photocatalytic efficiency, degrading 92.8% of the target molecules after 90 min of UV-light irradiation, compared to pristine Ti_3_C_2_-MXene nanosheets (31.3%) and the binary composite Ti_3_C_2_-MXene/NiO (62.9%). The Ti_3_C_2_-MXene/NiO/CsPbI_3_ composite also showed the highest rate constant (0.0199 min^−1^) and improved photocatalytic activity due to the reduction in band gap and strong synergistic effects at the interface between Ti_3_C_2_-MXene/NiO and CsPbI_3_. The addition of CsPbI_3_ enhanced the transport and separation of photogenerated electron–hole pairs at the NiO and Ti_3_C_2_-MXene interface. Therefore, the formation of Ti_3_C_2_-MXene/NiO/CsPbI_3_ composites involving perovskite materials and carbon-based materials proved to be an effective approach for removing organic pollutants from water under UV light. The improved performance, attributed to the larger surface area and lower electron–hole recombination rate, makes Ti_3_C_2_-MXene/NiO/CsPbI_3_ composites a highly efficient photocatalyst with promising applications in various fields.

## Data Availability

The data presented in this study are available upon request from the corresponding author.

## References

[1] Chu E.W., Karr J.R. (2017). Environmental Impact: Concept, Consequences, Measurement. Reference Module in Life Sciences.

[2] Akinnawo S.O. (2023). Eutrophication: Causes, Consequences, Physical, Chemical and Biological Techniques for Mitigation Strategies. Environ. Chall..

[3] Afrin S., Shuvo H.R., Sultana B., Islam F., Rus’d A.A., Begum S., Hossain M.N. (2021). The Degradation of Textile Industry Dyes Using the Effective Bacterial Consortium. Heliyon.

[4] Benkhaya S., M’rabet S., El Harfi A. (2020). Classifications, Properties, Recent Synthesis and Applications of Azo Dyes. Heliyon.

[5] Shabir M., Yasin M., Hussain M., Shafiq I., Akhter P., Nizami A.S., Jeon B.H., Park Y.K. (2022). A Review on Recent Advances in the Treatment of Dye-Polluted Wastewater. J. Ind. Eng. Chem..

[6] Gómez-Gómez A.L., Martínez-Ayala A.L., Moguel-Concha D.d.R., Borges-Martínez J.E., Perea-Flores M.d.J., Dávila-Ortiz G. (2023). Relationship of Nanomaterials’ Structure Based on Their Application in the Food Industry: Physicochemical and Techno-Functional Characteristics. Appl. Sci..

[7] Li X., Bai Y., Shi X., Su N., Nie G., Zhang R., Nie H., Ye L. (2021). Applications of MXene (Ti3C2T: X) in Photocatalysis: A Review. Mater. Adv..

[8] Yu H., Jiang H., Zhang S., Feng X., Yin S., Zhao W. (2023). Review of Two-Dimensional MXenes (Ti3C2Tx) Materials in Photocatalytic Applications. Processes.

[9] Amrillah T., Supandi A.R., Puspasari V., Hermawan A., Seh Z.W. (2022). MXene-Based Photocatalysts and Electrocatalysts for CO_2_ Conversion to Chemicals. Trans. Tianjin Univ..

[10] Raizada P., Soni V., Kumar A., Singh P., Parwaz Khan A.A., Asiri A.M., Thakur V.K., Nguyen V.H. (2021). Surface Defect Engineering of Metal Oxides Photocatalyst for Energy Application and Water Treatment. J. Mater..

[11] Park H., Son N., Park B.H., Joo S.W., Kang M. (2021). Visible Light-Induced Stable HER Performance Using Duality of Ultrafine Pt NPs in a Z-Scheme p-n Junction Fe2O3@Pt@FeS Catalyst. Appl. Surf. Sci..

[12] Taeño M., Maestre D., Cremades A. (2021). An Approach to Emerging Optical and Optoelectronic Applications Based on NiO Micro- and Nanostructures. Nanophotonics.

[13] Kumari H., Sonia, Suman, Ranga R., Chahal S., Devi S., Sharma S., Kumar S., Kumar P., Kumar S. (2023). A Review on Photocatalysis Used for Wastewater Treatment: Dye Degradation. Water Air Soil Pollut..

[14] Huang H., Pradhan B., Hofkens J., Roeffaers M.B.J., Steele J.A. (2020). Solar-Driven Metal Halide Perovskite Photocatalysis: Design, Stability, and Performance. ACS Energy Lett..

[15] Fu H., Liu X., Fu J., Wu Y., Zhang Q., Wang Z., Liu Y., Zheng Z., Cheng H., Dai Y. (2023). 2D/Quasi-2D Ruddlesden—Popper Perovskite: A High-Performance Photocatalyst for Hydrogen Evolution. ACS Catal..

[16] Drozdowski D., Gągor A., Stefańska D., Zarȩba J.K., Fedoruk K., Mączka M., Sieradzki A. (2022). Three-Dimensional Methylhydrazinium Lead Halide Perovskites: Structural Changes and Effects on Dielectric, Linear, and Nonlinear Optical Properties Entailed by the Halide Tuning. J. Phys. Chem. C.

[17] Shamsi J., Urban A.S., Imran M., De Trizio L., Manna L. (2019). Metal Halide Perovskite Nanocrystals: Synthesis, Post-Synthesis Modifications, and Their Optical Properties. Chem. Rev..

[18] Temerov F., Baghdadi Y., Rattner E., Eslava S. (2022). A Review on Halide Perovskite-Based Photocatalysts: Key Factors and Challenges. ACS Appl. Energy Mater..

[19] Luo J., Zhang W., Yang H., Fan Q., Xiong F., Liu S., Li D.S., Liu B. (2021). Halide Perovskite Composites for Photocatalysis: A Mini Review. EcoMat.

[20] Dandia A., Saini P., Sharma R., Parewa V. (2020). Visible Light Driven Perovskite-Based Photocatalysts: A New Candidate for Green Organic Synthesis by Photochemical Protocol. Curr. Res. Green Sustain. Chem..

[21] Zhang X., Liu A., Cao Y., Xie J., Jia W., Jia D. (2019). Interstitial N-Doped SrSnO_3_ Perovskite: Structural Design, Modification and Photocatalytic Degradation of Dyes. New J. Chem..

[22] Chen C., Zhou J., Geng J., Bao R., Wang Z., Xia J., Li H. (2020). Perovskite LaNiO_3_/TiO_2_ Step-Scheme Heterojunction with Enhanced Photocatalytic Activity. Appl. Surf. Sci..

[23] Kitchamsetti N., Didwal P.N., Mulani S.R., Patil M.S., Devan R.S. (2021). Photocatalytic Activity of MnTiO_3_ Perovskite Nanodiscs for the Removal of Organic Pollutants. Heliyon.

[24] Lele N., Bambo M.F., Mmutlane E.M., Dlamini L.N. (2023). Construction of a Multifunctional MXene@β-Cyclodextrin Nanocomposite with Photocatalytic Properties. Emergent Mater..

[25] Tan L., Lv J., Xu X., Zhao H., He C., Wang H., Zheng W. (2019). Construction of MXene/NiO Composites through in-Situ Precipitation Strategy for Dispersibility Improvement of NiO Nanoparticles. Ceram. Int..

[26] Wang P., Zhang X., Zhou Y., Jiang Q., Ye Q., Chu Z., Li X., Yang X., Yin Z., You J. (2018). Solvent-Controlled Growth of Inorganic Perovskite Films in Dry Environment for Efficient and Stable Solar Cells. Nat. Commun..

[27] Karthikeyan P., Elanchezhiyan S.S., Preethi J., Talukdar K., Meenakshi S., Park C.M. (2021). Two-Dimensional (2D) Ti_3_C_2_T_x_ MXene Nanosheets with Superior Adsorption Behavior for Phosphate and Nitrate Ions from the Aqueous Environment. Ceram. Int..

[28] Chavan R.A., Kamble G.P., Dhavale S.B., Rasal A.S., Kolekar S.S., Chang J.Y., Ghule A.V. (2023). NiO@MXene Nanocomposite as an Anode with Enhanced Energy Density for Asymmetric Supercapacitors. Energy Fuels.

[29] Shu M., Li R., Zhang Z. (2021). CsPbI_3_ Quantum Dots/Polypyrrole Microrod 0D/1D Heterostructure: Synthesis, Formation Mechanism and Enhanced Charge Transport Property. Mater. Chem. Phys..

[30] Mahmood M., Rasheed A., Ayman I., Rasheed T., Munir S., Ajmal S., Agboola P.O., Warsi M.F., Shahid M. (2021). Synthesis of Ultrathin MnO_2_nanowire-Intercalated 2D-MXenes for High-Performance Hybrid Supercapacitors. Energy Fuels.

[31] Zhang K., Ying G., Liu L., Ma F., Su L., Zhang C., Wu D., Wang X., Zhou Y. (2019). Three-Dimensional Porous Ti_3_C_2_ Tx-NiO Composite Electrodes with Enhanced Electrochemical Performance for Supercapacitors. Materials.

[32] Shen Q., Ripolles T.S., Even J., Ogomi Y., Nishinaka K., Izuishi T., Nakazawa N., Zhang Y., Ding C., Liu F. (2017). Slow Hot Carrier Cooling in Cesium Lead Iodide Perovskites. Appl. Phys. Lett..

[33] Kiran N.U., Deore A.B., More M.A., Late D.J., Rout C.S., Mane P., Chakraborty B., Besra L., Chatterjee S. (2022). Comparative Study of Cold Electron Emission from 2D Ti_3_C_2_TXMXene Nanosheets with Respect to Its Precursor Ti_3_SiC_2_MAX Phase. ACS Appl. Electron. Mater..

[34] Huang B., Tong X., Zhang X., Feng Q., Rumyantseva M.N., Prakash J., Li X. (2023). MXene/NiO Composites for Chemiresistive-Type Room Temperature Formaldehyde Sensor. Chemosensors.

[35] Zhang Q., Tai M., Zhou Y., Zhou Y., Wei Y., Tan C., Wu Z., Li J., Lin H. (2020). Enhanced Photocatalytic Property of γ-CsPbI_3_ Perovskite Nanocrystals with WS_2_. ACS Sustain. Chem. Eng..

[36] Aleksanyan E., Aprahamian A., Mukasyan A.S., Harutyunyan V., Manukyan K.V. (2020). Mechanisms of Mechanochemical Synthesis of Cesium Lead Halides: Pathways toward Stabilization of α-CsPbI_3_. J. Mater. Sci..

[37] Gull S., Batool S., Li G., Idrees M. (2022). Synthesis of Cesium Lead Halide Perovskite/Zinc Oxide (CsPbX_3_/ZnO, X = Br, I) as Heterostructure Photocatalyst with Improved Activity for Heavy Metal Degradation. Front. Chem..

[38] Ding C., Chen X., Zhang T., Zhou C., Liu X., Wang J., Lin J., Chen X. (2021). Electrochemical Synthesis of Annealing-Free and Highly Stable Black-Phase CsPbI_3_perovskite. Chem. Commun..

[39] Luo Q., Chai B., Xu M., Cai Q. (2018). Preparation and Photocatalytic Activity of TiO_2_-Loaded Ti_3_C_2_ with Small Interlayer Spacing. Appl. Phys. A Mater. Sci. Process..

[40] El-Kemary M., Nagy N., El-Mehasseb I. (2013). Nickel Oxide Nanoparticles: Synthesis and Spectral Studies of Interactions with Glucose. Mater. Sci. Semicond. Process..

[41] Iqbal M.A., Tariq A., Zaheer A., Gul S., Ali S.I., Iqbal M.Z., Akinwande D., Rizwan S. (2019). Ti_3_C_2_-MXene/Bismuth Ferrite Nanohybrids for Efficient Degradation of Organic Dyes and Colorless Pollutants. ACS Omega.

[42] Iqbal M.A., Ali S.I., Amin F., Tariq A., Iqbal M.Z., Rizwan S. (2019). La-and Mn-Codoped Bismuth Ferrite/Ti_3_C_2_ MXene Composites for Efficient Photocatalytic Degradation of Congo Red Dye. ACS Omega.

[43] Khan J., Ahmad R.T.M., Yu Q., Liu H., Khan U., Liu B. (2023). A La_2_O_3_/MXene Composite Electrode for Supercapacitors with Improved Capacitance and Cycling Performance. Sci. Technol. Adv. Mater..

[44] Li X., Wen C., Yuan M., Sun Z., Wei Y., Ma L., Li H., Sun G. (2020). Nickel Oxide Nanoparticles Decorated Highly Conductive Ti_3_C_2_ MXene as Cathode Catalyst for Rechargeable Li–O_2_ Battery. J. Alloys Compd..

[45] Neampet S., Ruecha N., Qin J., Wonsawat W., Chailapakul O., Rodthongkum N. (2019). A Nanocomposite Prepared from Platinum Particles, Polyaniline and a Ti_3_C_2_ MXene for Amperometric Sensing of Hydrogen Peroxide and Lactate. Microchim. Acta.

[46] Sekar S., Bathula C., Rabani I., Lee J.W., Lee S.H., Seo Y.S., Lee S. (2022). Enhanced Photocatalytic Crystal-Violet Degradation Performances of Sonochemically-Synthesized AC-CeO_2_ Nanocomposites. Ultrason. Sonochem..

[47] Le V.T., Doan V.D., Le T.T.N., Dao M.U., Vo T.T.T., Do H.H., Viet D.Q., Tran V.A. (2021). Efficient Photocatalytic Degradation of Crystal Violet under Natural Sunlight Using Fe3O4/ZnO Nanoparticles Embedded Carboxylate-Rich Carbon. Mater. Lett..

[48] Alsafari I.A., Munir S., Zulfiqar S., Saif M.S., Warsi M.F., Shahid M. (2021). Synthesis, Characterization, Photocatalytic and Antibacterial Properties of Copper Ferrite/MXene (CuFe_2_O_4_/Ti_3_C_2_) Nanohybrids. Ceram. Int..

[49] Sun B., Tao F., Huang Z., Yan W., Zhang Y., Dong X., Wu Y., Zhou G. (2021). Ti_3_C_2_ MXene-Bridged Ag/Ag_3_PO_4_ Hybrids toward Enhanced Visible-Light-Driven Photocatalytic Activity. Appl. Surf. Sci..

[50] Chaiwichian S., Wetchakun K., Kangwansupamonkon W., Wetchakun N. (2017). Novel Visible-Light-Driven BiFeO_3_-Bi_2_WO_6_ Nanocomposites toward Degradation of Dyes. J. Photochem. Photobiol. A Chem..

[51] Qamar S., Fatima K., Ullah N., Akhter Z., Waseem A., Sultan M. (2022). Recent Progress in Use of MXene in Perovskite Solar Cells: For Interfacial Modification, Work-Function Tuning and Additive Engineering. Nanoscale.

[52] Song T., Hou L., Long B., Ali A., Deng G.J. (2021). Ultrathin MXene “Bridge” to Accelerate Charge Transfer in Ultrathin Metal-Free 0D/2D Black Phosphorus/g-C_3_N_4_ Heterojunction toward Photocatalytic Hydrogen Production. J. Colloid Interface Sci..

[53] Zhu H., Fu X., Zhou Z. (2022). 3D/2D Heterojunction of CeO_2_/Ultrathin MXene Nanosheets for Photocatalytic Hydrogen Production. ACS Omega.

[54] Sanakousar M.F., Vidyasagar C.C., Jiménez-Pérez V.M., Jayanna B.K., Mounesh, Shridhar A.H., Prakash K. (2021). Efficient Photocatalytic Degradation of Crystal Violet Dye and Electrochemical Performance of Modified MWCNTs/Cd-ZnO Nanoparticles with Quantum Chemical Calculations. J. Hazard. Mater. Adv..

[55] Filipe O.M.S., Santos E.B.H., Otero M., Gonçalves E.A.C., Neves M.G.P.M.S. (2020). Photodegradation of Metoprolol in the Presence of Aquatic Fulvic Acids. Kinetic Studies, Degradation Pathways and Role of Singlet Oxygen, OH Radicals and Fulvic Acids Triplet States. J. Hazard. Mater..

[56] Nosaka Y., Nosaka A. (2016). Understanding Hydroxyl Radical (•OH) Generation Processes in Photocatalysis. ACS Energy Lett..

[57] Zhou Q., Zhang L., Zuo P., Wang Y., Yu Z. (2018). Enhanced Photocatalytic Performance of Spherical BiOI/MnO_2_ Composite and Mechanism Investigation. RSC Adv..

[58] Yamashita H., Ichihashi Y., Zhang S.G., Matsumura Y., Souma Y., Tatsumi T., Anpo M. (1997). Photocatalytic Decomposition of NO at 275 K on Titanium Oxide Catalysts Anchored within Zeolite Cavities and Framework. Appl. Surf. Sci..

[59] Li C., Kan C., Meng X., Liu M., Shang Q., Yang Y., Wang Y., Cui X. (2022). Self-Assembly 2D Ti_3_C_2_/g-C_3_N_4_ MXene Heterojunction for Highly Efficient Photocatalytic Degradation of Tetracycline in Visible Wavelength Range. Nanomaterials.

[60] Murali G., Reddy Modigunta J.K., Park Y.H., Lee J.H., Rawal J., Lee S.Y., In I., Park S.J. (2022). A Review on MXene Synthesis, Stability, and Photocatalytic Applications. ACS Nano.

[61] Liu C., Xiao W., Liu X., Wang Q., Hu J., Zhang S., Xu J., Zhang Q., Zou Z. (2023). Rationally Designed Ti_3_C_2_ MXene/CaIn_2_S_4_ Schottky Heterojunction for Enhanced Photocatalytic Cr(VI) Reduction: Performance, Influence Factors and Mechanism. J. Mater. Sci. Technol..

[62] Tahir M., Khan A.A., Tasleem S., Mansoor R., Sherryna A., Tahir B. (2023). Recent Advances in Titanium Carbide MXene-Based Nanotextures with Influential Effect of Synthesis Parameters for Solar CO_2_ Reduction and H2 Production: A Critical Review. J. Energy Chem..

[63] Liu C., Xiao W., Yu G., Wang Q., Hu J., Xu C., Du X., Xu J., Zhang Q., Zou Z. (2023). Interfacial Engineering of Ti_3_C_2_ MXene/CdIn_2_S_4_ Schottky Heterojunctions for Boosting Visible-Light H_2_ Evolution and Cr(VI) Reduction. J. Colloid Interface Sci..

[64] Chinnasamy C., Perumal N., Choubey A., Rajendran S. (2023). Recent Advancements in MXene-Based Nanocomposites as Photocatalysts for Hazardous Pollutant Degradation—A Review. Environ. Res..

[65] Zhou P., Navid I.A., Ma Y., Xiao Y., Wang P., Ye Z., Zhou B., Sun K., Mi Z. (2023). Solar-to-Hydrogen Efficiency of More than 9% in Photocatalytic Water Splitting. Nature.

[66] Liu C., Zhang Q., Zou Z. (2023). Recent Advances in Designing ZnIn2S4-Based Heterostructured Photocatalysts for Hydrogen Evolution. J. Mater. Sci. Technol..

[67] Sepehrmansourie H., Alamgholiloo H., Noroozi Pesyan N., Zolfigol M.A. (2023). A MOF-on-MOF Strategy to Construct Double Z-Scheme Heterojunction for High-Performance Photocatalytic Degradation. Appl. Catal. B Environ..

[68] Liu W., Wang Y., Huang H., Wang J., He G., Feng J., Yu T., Li Z., Zou Z. (2023). Spatial Decoupling of Redox Chemistry for Efficient and Highly Selective Amine Photoconversion to Imines. J. Am. Chem. Soc..

[69] Xia J., Sohail M., Nazeeruddin M.K. (2023). Efficient and Stable Perovskite Solar Cells by Tailoring of Interfaces. Adv. Mater..

[70] Saranin D., Pescetelli S., Pazniak A., Rossi D., Liedl A., Yakusheva A., Luchnikov L., Podgorny D., Gostischev P., Didenko S. (2021). Transition Metal Carbides (MXenes) for Efficient NiO-Based Inverted Perovskite Solar Cells. Nano Energy.

[71] Vijayakumar E., Govinda Raj M., Narendran M.G., Preetha R., Mohankumar R., Neppolian B., John Bosco A. (2022). Promoting Spatial Charge Transfer of ZrO_2_Nanoparticles: Embedded on Layered MoS_2_/g-C_3_N_4_Nanocomposites for Visible-Light-Induced Photocatalytic Removal of Tetracycline. ACS Omega.

